# Ontogeny of hepatic metabolism in two broiler lines divergently selected for the ultimate pH of the *Pectoralis major* muscle

**DOI:** 10.1186/s12864-024-10323-0

**Published:** 2024-05-02

**Authors:** Angélique Petit, Sophie Tesseraud, Anne Collin, Nathalie Couroussé, Cécile Berri, Elisabeth Le Bihan-Duval, Sonia Métayer-Coustard

**Affiliations:** INRAE, Université de Tours, BOA, Nouzilly, 37380 France

**Keywords:** Chicken, Embryo, Liver, Metabolism, Ontogeny, Gene expression

## Abstract

**Background:**

Nutrient availability during early stages of development (embryogenesis and the first week post-hatch) can have long-term effects on physiological functions and bird metabolism. The embryo develops in a closed structure and depends entirely on the nutrients and energy available in the egg. The aim of this study was to describe the ontogeny of pathways governing hepatic metabolism that mediates many physiological functions in the pHu + and pHu- chicken lines, which are divergently selected for the ultimate pH of meat, a proxy for muscle glycogen stores, and which differ in the nutrient content and composition of eggs.

**Results:**

We identified eight clusters of genes showing a common pattern of expression between embryonic day 12 (E12) and day 8 (D8) post-hatch. These clusters were not representative of a specific metabolic pathway or function. On E12 and E14, the majority of genes differentially expressed between the pHu + and pHu- lines were overexpressed in the pHu + line. Conversely, the majority of genes differentially expressed from E18 were overexpressed in the pHu- line. During the metabolic shift at E18, there was a decrease in the expression of genes linked to several metabolic functions (e.g. protein synthesis, autophagy and mitochondrial activity). At hatching (D0), there were two distinct groups of pHu + chicks based on hierarchical clustering; these groups also differed in liver weight and serum parameters (e.g. triglyceride content and creatine kinase activity). At D0 and D8, there was a sex effect for several metabolic pathways. Metabolism appeared to be more active and oriented towards protein synthesis (*RPS6*) and fatty acid β-oxidation (*ACAA2*, *ACOX1*) in males than in females. In comparison, the genes overexpressed in females were related to carbohydrate metabolism (*SLC2A1, SLC2A12*, *FoxO1*, *PHKA2*, *PHKB*, *PRKAB2* and *GYS2*).

**Conclusions:**

Our study provides the first detailed description of the evolution of different hepatic metabolic pathways during the early development of embryos and post-hatching chicks. We found a metabolic orientation for the pHu + line towards proteolysis, glycogen degradation, ATP synthesis and autophagy, likely in response to a higher energy requirement compared with pHu- embryos. The metabolic orientations specific to the pHu + and pHu- lines are established very early, probably in relation with their different genetic background and available nutrients.

**Supplementary Information:**

The online version contains supplementary material available at 10.1186/s12864-024-10323-0.

## Background

The perinatal period is a critical stage during which the environment plays a major role, with potential long-term effects on animal performance, robustness, health and welfare. This is a phase of metabolic plasticity, with the possibility of metabolic reorientation and early phenotype programming, notably through nutrition [1–3]. In oviparous vertebrates, such as birds, embryonic development takes place entirely in the egg. Therefore, embryos are dependent on the nutrient content of the egg, including energy sources (lipids and proteins), until they hatch [4]. Carbohydrates represent less than 1% and free glucose only 0.3% of the total nutrients. As hatching approaches, a reduced oxygen supply has a negative impact on energy production from fatty acid oxidation. Thus, glycogen stores are mobilised to maintain glucose homeostasis [5]. Given the importance of energy stores during the perinatal period [6, 7], we used an original model of two divergent lines for the ultimate pH of meat (pHu + and pHu-), reflecting muscle glycogen stores [8, 9]. In these lines, the main sources of nutrients in the egg differ in composition and/or nutrient content (e.g. lipids in yolk and branched-chain amino acids in amniotic fluid) [10]. We also observed early metabolic signatures (from embryonic day 10 [E10]) by studying the allantoic fluid metabolome, which indirectly reflects embryo metabolism [11]. In pHu + and pHu- lines, we showed that muscle metabolic differences, in particular the glycogen content, induced by the selection at slaughter age, are already present at hatching and amplified by the exogenous supply of nutrients during the early post-hatching stage [12]. In pHu lines, we explored mainly protein and carbohydrate metabolisms specifically in muscle but we have never explored more widely the orientation of their metabolism at the hepatic level whereas the liver is the crossroads of several metabolism.

The liver is an essential metabolic organ that performs exocrine and endocrine metabolic functions such as bile production, blood detoxification, regulation of circulating hormones, protein synthesis and regulation of glucose levels through glycogen storage [13]. In chickens, the liver is also the main site of *de novo* lipogenesis [14]. These functions depend on a complex hepatic vascular network that is established throughout embryogenesis, relying on both angiogenesis and vasculogenesis [15]. Blood from the yolk circulation, digestive tract or peripheral tissues passes through the liver, where nutrients can be absorbed, stored, modified or exported to other parts of the body, and waste products from other tissues can be metabolised for excretion or recycled into useful metabolites [16]. According to Désert et al. [17], the liver contributes to the adaptation of birds to changes in energy and nutrient sources, at least at the transcriptional level. There is also evidence that major changes in liver gene expression occur during the transition between the embryonic and post-hatching periods. However, studies have mainly focused on lipid and carbohydrate metabolism and have often overlooked the hatching process [5, 18–22].

In the present study, we aimed to understand how the main pathways and functions governing hepatic metabolism are set up, during early development, in response to the available nutrients and energy substrates, which evolve over time but also between the pHu + and pHu- lines [10, 11]. We established the kinetics at E12, E14, E18, hatching (day 0 [D0]) and day 8 post-hatch (D8). E12 corresponds to the stage at which the liver weight was sufficient for sampling. From E12 to E18, there is dynamic embryonic development during which the nutrient composition of the egg is likely to influence embryo metabolism and growth. Hatching is an energy-intensive stage that is accompanied by dramatic physiological and metabolic changes. We chose D8 to assess the impact of an exogenous nutrient supply during the first week post-hatch.

## Results

### Evolution of the embryo weight and the relative liver weight

**Fig. 1:**
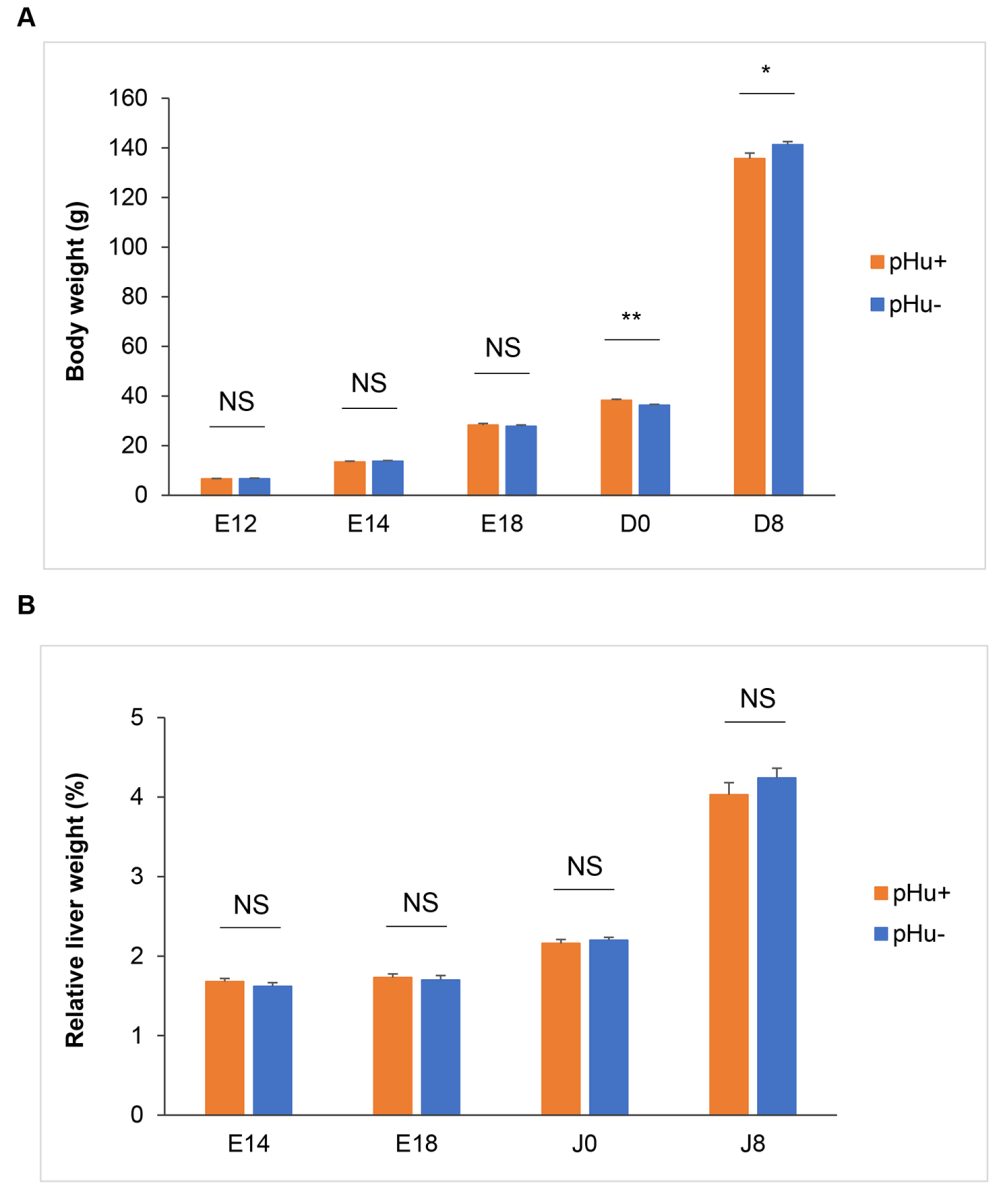
Evolution of animal body weight (**A**) and relative liver weight (**B**) from E12 to D8. The orange colour corresponds to the pHu + line and the blue colour to the pHu- line. *N* = 23–24 embryos per line at E12 and E14; *N* = 16 embryos per line at E18; *N* = 20 chicks per line at D0; *N* = 13 pHu + and 14 pHu- at D8. The data are presented at the mean ± standard error of the mean. Comparisons of means were analysed with Student’s t-test. NS = not significant; *** *P* ≤ 0.001; ** *P* ≤ 0.01; * *P* ≤ 0.05

The embryo weight increased significantly throughout development, with a more pronounced effect between D0 and D8 (Fig. 1A). In comparison, the relative liver weight was similar at E14 and E18, then increased at D0 and D8 (Fig. 1B). At hatching (D0), pHu + chicks were significantly heavier than pHu- chicks (*P* = 0.001), while at D8, pHu- chicks were heavier than pHu + chicks (*P* = 0.04). There was no line effect on embryo weight at E12, E14 and E18 or over the entire analysed development times for the relative liver weight.

### Changes in the expression of genes involved in hepatic metabolism of the pHu + and pHu- lines from E12 to D8

**Fig. 2:**
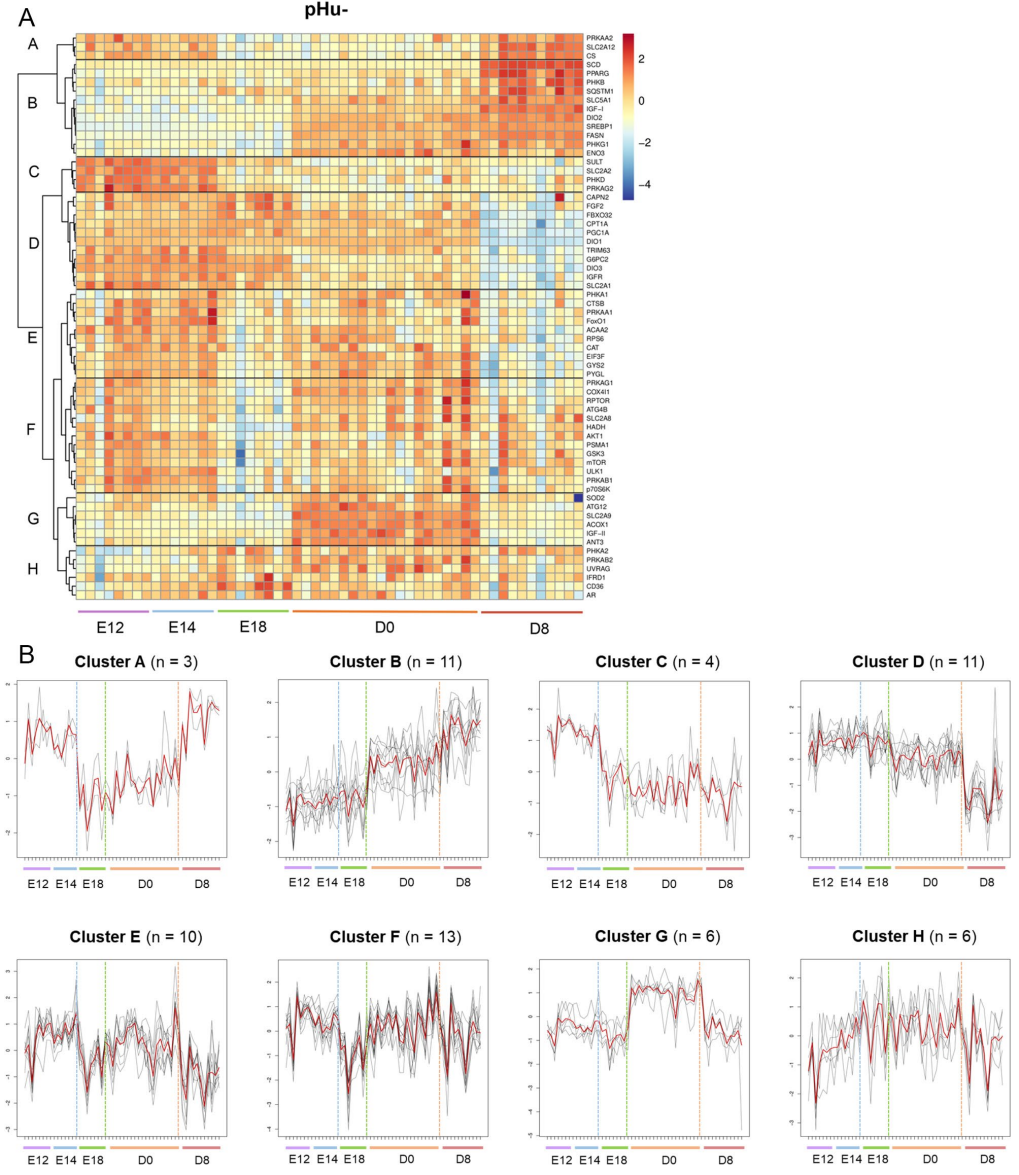
Changes in the expression of genes involved in hepatic pHu- metabolism from E12 to D8. The genes on the heatmap were clustered by using correlation as a distance measure (**A**). The letters (A to H) correspond to the eight expression profiles observed. The colour gradient from yellow to red indicates overexpression, while the colour gradient from yellow to blue indicates underexpression. The average profile obtained for each cluster is illustrated by a red line (**B**). The dashed lines on the x-axis correspond to the samples. The dotted lines in blue, green and orange show the different stages. The black curves represent genes belonging to the cluster. Cluster A = 3 genes; cluster B = 11 genes; cluster C = 4 genes; cluster D = 11 genes; cluster E = 10 genes; cluster F = 13 genes; cluster G = 6 genes; cluster H = 6 genes. *N* = 8 pools of three embryos at E12; *N* = 7 pools of three embryos at E14; *N* = 8 pools of two embryos at E18; *N* = 20 chicks at D0; *N* = 11 chicks at D8

**Fig. 3:**
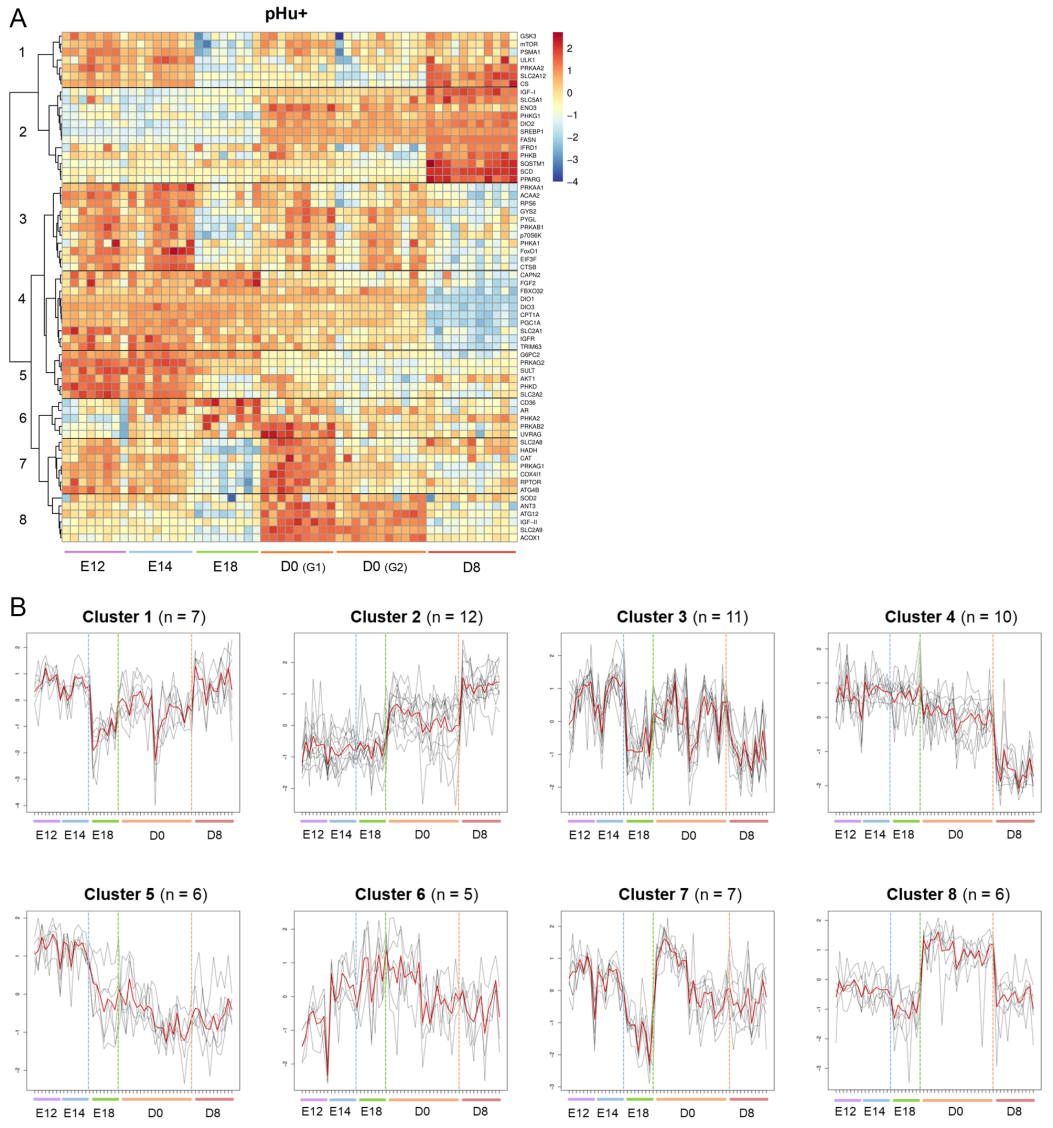
Changes in the expression of genes involved in hepatic pHu + metabolism from E12 to D8. Genes on the heatmap were clustered by using correlation as a distance measure (**A**). The numbers (1 to 8) correspond to the eight expression profiles observed. The colour gradient from yellow to red indicates overexpression, while the colour gradient from yellow to blue indicates underexpression. G1 and G2 correspond to the two groups of pHu + chicks for which differences were observed at hatching. The average profile obtained for each cluster is illustrated by a red line (**B**). The dashed lines on the x-axis correspond to the samples. The dotted lines in blue, green and orange show the different stages. The black curves represent genes belonging to the cluster. Cluster 1 = 7 genes; cluster 2 = 12 genes; cluster 3 = 11 genes; cluster 4 = 10 genes; cluster 5 = 6 genes; cluster 6 = 5 genes; cluster 7 = 7 genes; cluster 8 = 6 genes. *N* = 8 pools of three embryos at E12 and E14; *N* = 8 pools of two embryos at E18; *N* = 20 chicks at D0; *N* = 11 chicks at D8

The expression kinetics of genes involved in growth and development; carbohydrate, lipid, protein or steroid metabolism; mitochondrial activity, autophagy, thyroid hormone regulation, the antioxidant system; and cell growth and differentiation (Additional Table 1) are presented as heatmaps for pHu- and pHu+ (Figs. 2 and 3, respectively). A modular height cut-off value in the hierarchical tree identified eight clusters of genes with distinct expression profiles during ontogeny in the pHu- and pHu + lines. The number of genes and the expression profile of each cluster are presented for pHu- and pHu + lines.

**Table 1:** Relative expression of genes for which there were differences between pHu + and pHu- livers at E12 and E14

Stage	Metabolic pathways	Gene	pHu+	pHu-	Line effect
**E12**	Protein metabolism	*PSMA1*	3.55 ± 0.11	3.16 ± 0.12	**0.03**
		*CAPN2*	2.75 ± 0.28	2.12 ± 0.21	0.09
		*RPTOR*	3.02 ± 0.16	2.72 ± 0.06	0.09
		*AKT1*	3.23 ± 0.08	2.95 ± 0.11	**0.05**
	Carbohydrate metabolism	*PRKAB2*	2.27 ± 0.07	2.01 ± 0.07	**0.02**
		*PRKAG1*	3.40 ± 0.07	3.13 ± 0.05	**0.008**
		*PHKA1*	2.97 ± 0.28	2.37 ± 0.15	0.08
		*PHKB*	2.30 ± 0.06	2.14 ± 0.06	0.08
		*PHKG1*	2.00 ± 0.08	1.80 ± 0.07	0.08
	Mitochondrial activity	*COX4I1*	2.98 ± 0.08	2.63 ± 0.07	**0.004**
		*CS*	4.13 ± 0.14	3.52 ± 0.21	**0.03**
		*ANT3*	2.13 ± 0.06	1.95 ± 0.04	**0.02**
	Autophagy	*SQSTM1*	1.52 ± 0.05	1.38 ± 0.04	**0.05**
	Growth - Development	*IGF2*	1.69 ± 0.06	1.51 ± 0.04	**0.03**
	Cell growth - Differentiation	*IFRD1*	2.04 ± 0.13	2.41 ± 0.07	**0.02**
**E14**	Protein metabolism	*PSMA1*	3.16 ± 0.08	2.88 ± 0.11	0.06
		*CAPN2*	3.34 ± 0.16	2.79 ± 0.11	**0.02**
		*RPTOR*	2.97 ± 0.05	2.62 ± 0.09	**0.004**
		*SLC2A9*	1.83 ± 0.10	1.42 ± 0.11	**0.01**
	Carbohydrate metabolism	*PRKAB2*	2.64 ± 0.04	2.20 ± 0.15	**0.008**
		*PRKAG1*	3.08 ± 0.09	2.85 ± 0.08	0.09
		*GSK3*	2.58 ± 0.04	2.43 ± 0.08	0.09
		*SLC2A1*	3.52 ± 0.10	3.08 ± 0.22	0.08
	Lipid metabolism	*PPARG*	1.16 ± 0.05	0.98 ± 0.03	**0.008**
		*CPT1A*	2.45 ± 0.06	1.87 ± 0.13	**0.001**
	Mitochondrial activity	*COX4I1*	2.78 ± 0.06	2.40 ± 0.09	**0.002**
		*ANT3*	2.18 ± 0.05	1.87 ± 0.07	**0.003**
		*PGC1A*	3.65 ± 0.20	3.06 ± 0.19	**0.05**
	Autophagy	*SQSTM1*	1.78 ± 0.07	1.47 ± 0.07	**0.009**
		*ATG4B*	2.72 ± 0.06	2.50 ± 0.07	**0.03**
	Antioxidant system	*SOD2*	1.83 ± 0.21	2.66 ± 0.19	**0.01**
	Cell growth - Differentiation	*IFRD1*	1.99 ± 0.07	2.37 ± 0.07	**0.002**

The data are presented as the mean ± standard error of the mean and are expressed in arbitrary units. Student’s t-test was used to compare the means. Values in bold indicate significant differences between pHu + and pHu- (*P* ≤ 0.05). The threshold value for a trend was *P* < 0.10. The gene names are listed in Additional Table 1

In the pHu- line (Fig. 2A and B), cluster A includes *PRKAA2* and *SLC2A12* (carbohydrate metabolism) and *CS* (mitochondrial activity). The average profile showed a relatively stable expression from E12 to E14, a decrease between E14 and E18 and then an increase between E18 and D8. Cluster B includes *PHKB*, *PHKG1*, *ENO3* and *SLC5A1* (carbohydrate metabolism); *SREBP1*, *PPARG*, *FASN* and *SCD* (lipid metabolism); *DIO2* (thyroid hormone regulation); *SQSTM1* (autophagy); and *IGF1* (growth and development). It was characterised by an average profile whose expression was weak and stable from E12 to E18 and then increased from hatching to reach a maximum at D8.

In contrast, cluster C regroups *PHKD*, *PRKAG2* and *SLC2A2* (carbohydrate metabolism) and *SULT* (catecholamine and steroid metabolism), whose expression decreased from E12–E14 to D8. Cluster D includes *CAPN2*, *FBXO32* and *TRIM63* (protein metabolism); *G6PC2* and *SLC2A1* (carbohydrate metabolism); *CPT1A* (lipid metabolism); *PGC1*A (mitochondrial biogenesis); *IGFR* (growth and development); *FGF2* (cell growth and differentiation); and *DIO1* and *DIO3* (thyroid hormone regulation), whose expression was quite stable from E12 to E18 before decreasing until D8. The decrease was more pronounced between D0 and D8 than between E18 and D0. Despite relatively similar profiles, cluster C showed an earlier decrease in gene expression (between E14 and E18) than cluster D (between E18 and D0).

Cluster E – which comprises *EIF3F*, *RPS6* and *CTSB* (protein metabolism); *PHKA1*, *PRKAA1*, *FOXO1*, *GYS2* and *PYGL* (carbohydrate metabolism); *ACAA2* (lipid metabolism); and *CAT* (antioxidant system) – differed from the other clusters by a succession of decreased and increased expression according to the developmental stage. There was a decrease in expression between E14 and E18 and between D0 and D8, and an increase between E18 and D0. Cluster F includes *AKT1*, *MTOR*, *RPTOR*, *P70S6K* and *PSMA1* (protein metabolism); *PRKAB1*, *PRKAG1*, *GSK3* and *SLC2A8* (carbohydrate metabolism); *HADH* (lipid metabolism); *COX4I1* (mitochondrial activity); and *ULK1* and *ATG4B* (autophagy). It had a relatively similar average profile to cluster E, except during the post-hatching period when expression levels were comparable at D0 and D8.

Cluster G that gathers *SLC2A9* (hexose and uric acid transport), *ACOX1* (lipid metabolism), *ANT3* (mitochondrial activity), *ATG12* (autophagy), *SOD2* (antioxidant system) and *IGF2* (growth and development) showed a peak in expression at D0 followed by a decline at D8 to reach values similar to those observed in the early stages. Finally, the expression *PHKA2*, *PRKAB2* (carbohydrate metabolism), *CD36* (lipid metabolism), *UVRAG* (autophagy), *AR* and *IFRD1* (cell growth and differentiation), belonging to cluster H, increased between E12 and D0 before decreasing.

The expression profiles of genes contained in clusters A, B, C, D, E, F, G and H observed in the pHu- line (Fig. 2A) correspond approximately and respectively to those of clusters 1, 2, 5, 4, 3, 7, 8 and 6 in the pHu + line (Fig. 3A). Most of the genes included in each cluster showed similar expression profiles between the two lines, except for those belonging to clusters A and F (Additional Table 2). We observed differences in expression between the lines for *PSMA1*, *GSK3*, *MTOR* and *ULK1* (cluster 1); *IFRD1* (cluster 2); *PRKAB1* and *P70S6K* (cluster 3); *G6PC2* and *AKT1* (cluster 5); and *CAT* (cluster 7).

**Table 2:** Relative expression of genes for which there were differences between pHu + and pHu- livers from E18 to D8

Stage	Metabolic pathways	Gene	pHu+	pHu-	Line effect
**E18**	Carbohydrate metabolism	*G6PC2*	3.94 ± 0.11	3.18 ± 0.22	**0.008**
		*FoxO1*	2.20 ± 0.10	2.56 ± 0.13	**0.05**
	Lipid metabolism	*ACOX1*	0.84 ± 0.03	1.07 ± 0.05	**0.001**
	Antioxidant system	*SOD2*	1.22 ± 0.19	1.98 ± 0.20	**0.02**
	Cell growth - Differentiation	*IFRD1*	2.23 ± 0.10	2.64 ± 0.17	**0.05**
**D0**	Carbohydrate metabolism	*PRKAB2*	3.10 ± 0.07	3.28 ± 0.07	0.08
		*PRKAG2*	1.48 ± 0.03	1.69 ± 0.03	**< 0.0001**
		*SLC2A8*	2.20 ± 0.04	2.41 ± 0.04	**0.0008**
	Lipid metabolism	*CPT1A*	1.52 ± 0.07	1.86 ± 0.12	**0.02**
		*HADH*	2.45 ± 0.06	2.63 ± 0.08	0.08
		*CD36*	2.59 ± 0.09	2.92 ± 0.10	**0.02**
		*SREBP1*	4.08 ± 0.17	3.29 ± 0.23	**0.008**
	Autophagy	*UVRAG*	2.40 ± 0.07	2.70 ± 0.11	**0.03**
	Antioxidant system	*SOD2*	2.53 ± 0.23	3.05 ± 0.19	0.09
	Cell growth - Differentiation	*IFRD1*	2.00 ± 0.10	2.50 ± 0.07	**0.0003**
**D8**	Protein metabolism	*SLC2A9*	1.54 ± 0.07	1.72 ± 0.05	**0.05**
	Carbohydrate metabolism	*PHKB*	3.04 ± 0.07	3.49 ± 0.10	**0.002**
		*PHKD*	1.94 ± 0.09	2.22 ± 0.12	0.08
		*PRKAA1*	2.14 ± 0.07	2.51 ± 0.16	**0.04**
		*PRKAG2*	1.62 ± 0.07	1.98 ± 0.13	**0.03**
		*SLC2A2*	1.88 ± 0.12	1.54 ± 0.06	**0.02**
		*FoxO1*	2.01 ± 0.06	2.28 ± 0.09	**0.02**
	Antioxidant system	*CAT*	2.07 ± 0.09	1.77 ± 0.06	**0.01**
	Thyroid hormone regulation	*DIO3*	0.03 ± 0.005	0.07 ± 0.02	**0.03**

The data are presented as the mean ± standard error of the mean and are expressed in arbitrary units. Student’s t-test was used to compare the means. Values in bold indicate significant differences between pHu + and pHu- (*P* ≤ 0.05). The threshold value for a trend was *P* < 0.10. The gene names are listed in Additional Table 1

### Differentially expressed genes in the liver of the pHu + and pHu- lines

Among the 64 genes studied in the liver (Additional Table 1), we used normalised data to examine the differences in gene expression between the pHu + and pHu- lines at each stage. Significantly differentially expressed genes (*P* < 0.05) or genes with a trend for differential expression (*P* < 0.10) between the two lines are shown in Table 1 – for E12 (*n* = 15) and E14 (*n* = 17) – and Table 2 – for E18 (*n* = 5), D0 (*n* = 10) and D8 (*n* = 9).

At E12 and E14, most differentially expressed genes were overexpressed in the pHu + line, except for *IFRD1* and *SOD2* that were overexpressed in the pHu- line (Table 1). At E12, the expression of *PSMA1*, *AKT1*, *PRKAB2*, *PRKAG1*, *COX4I1*, *CS*, *ANT3*, *SQSTM1* and *IGF2* was significantly higher in the pHu + line than in the pHu- line (with trends for *CAPN2*, *RPTOR*, *PHKA1*, *PHKB* and *PHKG1*). At E14, the expression of *CAPN2*, *RPTOR*, *SLC2A9*, *PRKAB2*, *PPARG*, *CPT1A*, *COX4I1*, *ANT3*, *PGC1A*, *SQSTM1* and *AT4B* was significantly higher in the pHu + line than in the pHu- line with trends for *PSMA1*, *PRKAG1*, *GSK3* and *SLC2A1*).

On the contrary, most of the significant differences and trends between E18 and D8 indicated overexpression in the pHu- line (Table 2). Indeed, we observed significantly higher expression of *FoxO1*, *ACOX1*, *SOD2* and *IFRD1* at E18; *PRKAG2*, *SLC2A8*, *CPT1A*, *CD36*, *UVRAG* and *IFRD1* at D0 (with trends for *PRKAB2*, *HADH* and *SOD2*); and *SLC2A9*, *PHKB*, *PRKAA1*, *PRKAG2*, *FoxO1* and *DIO3* at D8 (with a trend for *PHKD*). Only *G6PC2* (E18), *SREBP1* (D0), *SLC2A2* and *CAT* (D8) were overexpressed in the pHu + line.

### Differentially expressed genes in the liver of males and females

Interestingly, we observed differential expression of several genes between male and female chicks. At hatching, *RPS6*, *PRKAA1*, *SLC2A8*, *PPARG*, *ACAA2*, *ATG12* and *DIO1* were overexpressed in males (with trends for *COX4I1* and *IGF2*), while only *PGC1A* was overexpressed in females (with trends for *SLC2A1* and *FoxO1*) (Table 3).

**Table 3:** Relative expression of genes for which there was a sex effect in the liver at D0

Metabolic pathways	Genes	Male	Female	Sex effect	
Protein metabolism	*RPS6*	2.99 ± 0.13	2.14 ± 0.10	**< 0.0001**	
Carbohydrate metabolism	*PRKAA1*	3.52 ± 0.19	2.85 ± 0.21	**0.02**	
	*SLC2A1*	2.36 ± 0.06	2.52 ± 0.07	0.09	
	*SLC2A8*	2.37 ± 0.04	2.23 ± 0.05	**0.03**	
	*FoxO1*	2.70 ± 0.14	3.13 ± 0.21	0.09	
Lipid metabolism	*PPARG*	1.71 ± 0.06	1.51 ± 0.05	**0.01**	
	*ACAA2*	3.32 ± 0.13	2.13 ± 0.11	**< 0.0001**	
Mitochondrial activity	*PGC1A*	2.14 ± 0.10	2.53 ± 0.16	**0.04**	
	*COX4I1*	3.08 ± 0.05	2.91 ± 0.07	0.06	
Autophagy	*ATG12*	6.62 ± 0.34	4.63 ± 0.26	**< 0.0001**	
Growth - development	*IGF2*	4.52 ± 0.29	3.86 ± 0.21	0.08	
Thyroid hormone regulation	*DIO1*	3.14 ± 0.06	2.81 ± 0.06	**0.0003**	

The data are presented as the mean ± standard error of the mean and are expressed in arbitrary units. Student’s t-test was used to compare the means. Values in bold indicate significant differences between pHu + and pHu- (*P* ≤ 0.05). The threshold value for a trend was *P* < 0.10. *N* = 20 chicks per line (10 females and 10 males). No line*sex interaction was noted. The gene names are listed in Additional Table 1

At D8, *RPS6*, *PRKAA1*, *ACAA2*, *ATG12* and *DIO1* were overexpressed in males (with trends for *SLC5A1* and *ACOX1*), while *PHKA2*, *PHKB* and *SLC2A12* were overexpressed in females (with trends for *PRKAB2* and *GYS2*) (Table 4). There was no line*sex interaction (data not shown), except for *SLC2A9* at D8, whose expression was significantly lower in pHu + males than in pHu- males and in pHu + and pHu-females.

**Table 4:** Relative expression of genes for which there was a sex effect in the liver at D8

Metabolic pathways	Genes	Male	Female	Sex effect
Protein metabolism	*RPS6*	2.49 ± 0.04	1.77 ± 0.09	**< 0.0001**
Carbohydrate metabolism	*PRKAA1*	2.52 ± 0.11	2.17 ± 0.13	**0.05**
	*PRKAB2*	2.30 ± 0.08	2.55 ± 0.11	0.09
	*PHKA2*	2.93 ± 0.16	3.32 ± 0.10	**0.04**
	*PHKB*	3.05 ± 0.10	3.44 ± 0.09	**0.007**
	*GYS2*	1.44 ± 0.10	1.72 ± 0.11	0.07
	*SLC5A1*	6.10 ± 0.74	4.78 ± 0.25	0.08
	*SLC2A12*	4.33 ± 0.31	5.45 ± 0.34	**0.03**
Lipid metabolism	*ACOX1*	1.59 ± 0.05	1.43 ± 0.06	0.07
	*ACAA2*	2.00 ± 0.11	1.36 ± 0.09	**0.0003**
Autophagy	*ATG12*	2.89 ± 0.15	2.23 ± 0.15	**0.005**
Thyroid hormone regulation	*DIO1*	2.72 ± 0.10	2.40 ± 0.11	**0.05**

The data are presented as the mean ± standard error of the mean and are expressed in arbitrary units. Student’s t-test was used to compare the means. Values in bold indicate significant differences between pHu + and pHu- (*P* ≤ 0.05). The threshold value for a trend was *P* < 0.10. *N* = 11 chicks per line (6 females and 5 males). No line*sex interaction was noted. The gene names are listed in Additional Table 1

### Phenotypic characterisation of the pHu- and pHu + lines at hatching

Based on the expression at hatching of genes belonging to clusters 1, 5, 6 and 7 (Fig. 3A), we were able to distinguish two subgroups of chicks (group 1 [G1] and group 2 [G2]) in the pHu + line but not in the pHu- line. When looking at their phenotypic differences, we observed that G1 exhibited a higher liver weight, triglyceride concentration and creatine kinase activity in their serum than G2 (Table 5). Regardless of the pHu + subgroup considered, at hatching pHu- chicks exhibited a lower body weight and serum creatine kinase activity. pHu- chicks had a lower liver weight than G1 pHu + chicks and a higher serum hydroxybutyrate concentration than G2 pHu + chicks. There was no significant difference between pHu- chicks and G1 and G2 pHu + chicks regarding residual yolk sac weight and yield, serum glucose, uric acid, the total antioxidant status and liver glycogen measured through the glycolytic potential. For all parameters studied, there was no sex effect or a group*sex interaction (data not shown).

**Table 5:** Comparison of phenotypic characteristics between pHu- and pHu+ (G1 and G2) at D0

	pHu-	pHu+ (G1)	pHu+ (G2)	*P*-Value
Chick weight (g)	36.3 ± 0.35 ^b^	38.8 ± 0.63 ^a^	37.8 ± 0.62 ^a^	**0.003**
Residual yolk sac weight (g)	2.75 ± 0.25	3.35 ± 0.29	3.18 ± 0.23	0.25
Liver weight (g)	0.80 ± 0.01 ^b^	0.88 ± 0.02 ^a^	0.78 ± 0.03 ^b^	**0.007**
Residual yolk sac / chick weight (%)	7.52 ± 0.65	8.60 ± 0.67	8.39 ± 0.59	0.48
Liver / chick weight (%)	2.20 ± 0.04 ^ab^	2.26 ± 0.04 ^a^	2.07 ± 0.07 ^b^	**0.05**
Glucose (mg/L)	1920 ± 23	1872 ± 43	1902 ± 34	0.57
Uric acid (mg/L)	68.9 ± 7.6	56.2 ± 4.7	73.0 ± 7.7	0.40
Triglycerides (mg/L)	490 ± 33 ^ab^	615 ± 81 ^a^	407 ± 46 ^b^	**0.04**
Non-esterified fatty acids (µmol/L)	380 ± 18	391 ± 26	323 ± 15	0.07
Hydroxybutyrate (mmol/L)	2.34 ± 0.09 ^a^	2.24 ± 0.17 ^ab^	1.88 ± 0.12 ^b^	**0.03**
Creatine kinase activity (UI/L)	4289 ± 857 ^c^	30,775 ± 7666 ^a^	16,233 ± 4600 ^b^	**< 0.0001**
Total antioxidant status (mmol/L)	1.06 ± 0.07	1.04 ± 0.06	1.11 ± 0.07	0.82
Liver glycolytic potential (µmol/g) *	108 ± 8	116 ± 13	97 ± 11	0.49

The phenotypic characterization includes weight, serum and tissue parameters. The data are presented as the mean ± standard error of the mean. The data are analyzed by ANOVA and multiple comparisons by a Tukey test. Values in bold indicate significant differences between pHu + and pHu- (*P* ≤ 0.05). Letters^a b c^ correspond to significant differences. The threshold value for a trend was *P* < 0.10. *N* = 20 chicks per line. *N* = 9 chicks in group 1 (G1) and *N* = 11 chicks in group 2 (G2), G1 and G2 being considered as two distinct groups of pHu + by hierarchical clustering of samples. * = equivalent lactate

At D8, pHu- chicks (both males and females) were significantly heavier and exhibited a higher serum hydroxybutyrate concentration than pHu + chicks (Table 6). At this age, pHu- chicks also tended to have a higher liver glycolytic potential than pHu + chicks (*P* = 0.06). There was a sex effect for the glucose concentration and relative liver weight: the glucose concentration was higher in males while the relative liver weight was higher in females. There was no line*sex interaction.

**Table 6:** Comparison of phenotypic characteristics between pHu- and pHu + at D8

	pHu+		pHu-		Line	Sex
	Male	Female	Male	Female		
Chick weight (g)	135 ± 4	136 ± 3	141 ± 3	141 ± 2	**0.04**	0.59
Liver weight (g)	5.2 ± 0.2	5.8 ± 0.4	5.7 ± 0.2	6.2 ± 0.3	0.10	0.06
Liver / chick weight (%)	3.8 ± 0.1	4.2 ± 0.3	4.0 ± 0.1	4.4 ± 0.2	0.28	**0.04**
Glucose (mg/L)	3839 ± 489	2683 ± 164	3874 ± 717	2712 ± 114	0.61	**0.005**
Uric acid (mg/L)	115 ± 11	98 ± 7	138 ± 13	115 ± 10	0.15	0.14
Triglycerides (mg/L)	2145 ± 293	2082 ± 179	2094 ± 244	2165 ± 115	0.91	0.97
Non-esterified fatty acids (µmol/L)	237.7 ± 12.7	241.4 ± 29.1	226.0 ± 13.3	226.2 ± 9.7	0.41	0.91
Hydroxybutyrate (mmol/L)	0.25 ± 0.06	0.25 ± 0.04	0.63 ± 0.12	0.40 ± 0.05	**0.006**	0.45
Creatine kinase activity (UI/L)	3257 ± 264	3075 ± 414	3488 ± 611	3725 ± 262	0.20	0.70
Total antioxidant status (mmol/L)	1.70 ± 0.12	1.57 ± 0.08	1.83 ± 0.11	1.60 ± 0.10	0.57	0.11
Liver glycolytic potential (µmol/g) *	1556 ± 88	1718 ± 113	1760 ± 94	1922 ± 75	0.06	0.15

The phenotypic characterization includes weight, serum and tissue parameters. The data are presented as the mean ± standard error of the mean. The data are analyzed by a two-way ANOVA. No significant interaction was observed. Values in bold indicate significant differences (*P* ≤ 0.05). The threshold value for a trend was *P* < 0.10. *N* = 14 pHu- (9 females and 5 males) and *N* = 13 pHu+ (6 females and 7 males). * = equivalent lactate

## Discussion

In fast-growing broilers, metabolic orientation can be established very early during embryonic development. This has been recently shown by Petit et al. [11], who observed at E10 specific metabolic signatures in the allantoic fluid of two experimental chicken lines (pHu + and pHu-) that were divergently selected for the ultimate pH of breast meat, a proxy of the muscle glycogen stores [8, 9]. The allantoic fluid is a compartment that stores nitrogenous waste products and metabolites and can reflect embryo protein–energy metabolic activity. To go further in the understanding of the mechanisms involved in early metabolic orientation of the pHu+/pHu- model, we followed the expression kinetics of a wide set of gene markers of several metabolic pathways and functions in the liver between E12 and D8. Although the transition between the embryonic and post-hatching periods is well documented in the chicken, studies have mainly focused on lipid and carbohydrate metabolism and have often overlooked the hatching process [5, 18–22]. In the present study, we first deeply described the expression profile of genes involved in protein, carbohydrate and lipid metabolism; mitochondrial activity; autophagy; antioxidant protection; thyroid hormone regulation; growth; and development from E12 to D8, and then explored the differences that could contribute to metabolic differences between the pHu + and the pHu- lines [12].

Although there were some differences in kinetics or gene expression, the general pattern of expression of most of the studied genes was very similar between the lines, which indicates that selection on the ultimate pH of the meat had no major effect on the establishment of metabolic pathways in the liver. Furthermore, we clearly showed that the different profiles are not representative of a specific metabolic pathway or function; rather, each cluster includes genes related to many functions.

E12 and E14 are characterised by very high expression of genes involved in protein (e.g. *AKT1*, *PSMA1* and *CTSB*) and carbohydrate (e.g. *FoxO1*, *PHKD*, *PRKAA1*, *G6PC2*, *SLC2A1* and *SLC2A2*) metabolism as well as fatty acid β-oxidation (e.g. *ACAA2* and *HADH*), mitochondrial activity (e.g. *CS*), autophagy (*ULK1* and *ATG4B*) and hormone metabolism (*SULT*). These results indicate that hepatic metabolism is very active at that time, a view that is consistent with the strong need of the embryo at a period when growth and metabolic rates are maximal and energy mainly provided by β-oxidation of fatty acids derived from yolk lipids [18, 23–25].

Between E14 and E18, the majority of genes involved in protein synthesis (*EIF3F*, *AKT1*, *MTOR*, *RPTOR*, *P70S6K* and *RPS6*), carbohydrate metabolism (PRKAA2, *PRKAG1*, *PYGL*, *GSK3*, *GYS2*, *SLC2A8* and *SLC2A12*), β-oxidation of medium- and short-chain fatty acids (*HADH*), mitochondrial activity (*CS*) and autophagy (*ULK1*, *ATG4B* and *ATG12*) showed a decrease in expression, suggesting a slowdown in hepatic metabolism at this stage that could be partly due to a reduction in available energy and the use of certain nutrients such as fatty acids *via* β-oxidation for energy purposes [4, 26].

The embryonic to post-hatching transition is characterised by major changes in liver gene expression. One of the most striking features concerns lipid metabolism with the increased expression of lipogenesis-related genes (*FASN*, *SREBP1*, *SCD* and *PPARG*) and decreased expression of lipolysis-related genes (*CPT1A* and *PGC1A*) as previously observed in chickens [18, 22, 27] and ducks [21]. While embryos use yolk lipids as the main energy substrate after the first week of incubation, hatched chick must switch to a diet rich in carbohydrates. Therefore, the post-hatching dietary transition is accompanied by major metabolic changes in the liver: it acquires the ability to synthesise its own lipids through *de novo* lipogenesis from carbohydrates [21, 28].

We also noted drastic changes in the expression of deiodinases during the embryonic to post-hatching transition, with a strong increase for *DIO2* and a decrease for *DIO3*, in agreement with previous results [18]. Deiodinases are key regulators of the intracellular thyroid hormone contents [29]. Triiodothyronine (T3) and thyroxine (T4) are related to heat production as well as glycogen and fat mobilisation. Thyroid hormones are also involved in the development of the pipping muscle and preparation for eggshell rupture [30]. Lu et al. [31] and Reyns et al. [32] reported that liver and plasma T3 levels were relatively low throughout the incubation period, with a significant increase at hatching that persisted through the first week post-hatch. Conversely, T4 levels increased significantly from E15 and reached a peak at E19. Our study showed very high expression of hepatic *DIO3* between E12 and E18. Because DIO3 converts T4 and T3 into inactive metabolites – rT3 and diiodothyronine (T2), respectively – DIO3 could play a major role in preventing premature exposure of developing embryonic tissues to high levels of thyroid hormone at this stage [33]. Between E18 and D8, we observed a constant decrease in *DIO3*, a transient increase in *DIO1* at hatching and a significant increase in *DIO2* in order to supply T3, the metabolically active thyroid hormone, to the chicks at hatching.

Hatching is also a key period during which a relay is established between the different insulin-like growth factors (IGFs) that play an important role in development and metabolism *via* autocrine/paracrine mechanisms. In the current study, the *IGF2* gene was expressed in the liver as early as E12 and showed a peak in expression at hatching. IGF2 is involved in embryogenesis and highly expressed in organs throughout the growth of the embryo [34]. On the contrary, the expression of *IGF1* remained very low during embryogenesis and increased considerably from D0 to D8, whereas the IGF receptor (IGFR) clearly decreased after hatching. Our results are consistent with previous studies that reported a strong increase in *IGF1* expression in the liver after hatching, with this organ being the main contributor of circulating IGF1 [35, 36].

Several genes showed a transient increase in expression at hatching. They are related to glycogen metabolism (*GYS2*, *PYGL*, *PHKA1*, *PRKAB1*, *PRKAB2* and *PRKAG1*), mammalian target of rapamycin (*MTOR*) signalling (*RPTOR* and *P70S6K*) and the cellular response to nutrient levels (*PRKAB*1, *PRKAB2*, *PRKAG1* and *RPTOR*) (Additional Fig. 1). We also found genes related to autophagy (*UVRAG*, *ATG12* and *ATG4B*), a lysosomal degradation pathway known to be an essential cellular metabolic process and a major contributor to homeostasis through the mobilisation of various cellular energy stores [37, 38], and genes that affect mitochondrial function (*COX4I1* and *ANT3*) by acting on oxidative phosphorylation and adenosine triphosphate (ATP) production. All these observations are consistent with the high energy demand associated with the hatching process. We also found a transient increase at hatching in the expression of redox status–regulating genes (*CAT*, *SOD2* and *ACOX1*) that likely contribute to the production of reactive oxygen species (ROS) and to the response to increased oxygen availability during this specific period. Moreover, the increased expression of genes related to purine metabolism (*SLC2A9*) and messenger RNA (mRNA)-to-protein translation (*P70S6K* and *EIF3F*) between E18 and D0 could indicate overactivated purine metabolism and protein synthesis, as already suggested by Peng et al. [39].

Although the evolution of gene expression in the liver during early development was quite similar between the pHu + and pHu- lines, our study highlighted some specificities related to each of them, some of which occurred as early as E12. It is worth noting that at the earliest stages studied (E12 and E14), most of the differentially expressed genes were overexpressed in the pHu + line, while from E18 onwards, most of the differentially expressed genes were overexpressed in the pHu- line. Among the differentially expressed genes at E12 and E14, the majority are involved in protein and carbohydrate metabolism and in processes linked to mitochondrial activity such as the Krebs cycle, the respiratory chain and adenosine diphosphate (ADP) and ATP transport. The glucose generated *via* glycogenolysis could have stimulated the Krebs cycle for ATP production, through the overexpression of mitochondrial genes (*CS*, *COX4I1* and *ANT3)* in the pHu + line. At E14, we observed a peak in *PGC1A* expression in the pHu + line, leading to an overexpression of this gene in this line compared with the pHu- line. *PGC1A* encodes a transcriptional coactivator that regulates genes involved in mitochondrial biogenesis, gluconeogenesis and fatty acid oxidation. This suggests a greater energy requirement during early embryonic development and, consequently, more intense metabolism to produce energy during this period in the pHu + line than in the pHu- line. The significant number of differentially expressed genes between the lines during the early stages of embryonic development indicates that the metabolic orientation switch between the two lines occurs as early as E12, with a major switch at E18, when the availability of nutrients and energy decreases.

At D0, 70% of the genes differentially expressed between the pHu + and pHu- lines were related to lipid or carbohydrate metabolism, while at D8, 67% of the genes were only related to carbohydrate metabolism. Similarly, to E18, at D0 the overexpression of genes linked to lipid metabolism and more specifically to fatty acid β-oxidation and transport (*ACOX1*, *CPT1A*, *HADH* and *CD36*) in the pHu- line could indicate a greater use of lipids in this line during the perinatal period. This view is consistent with the weight of the residual yolk sac, which tended to be higher in the pHu + line compared with the pHu- line, although the difference was not significant. At D8, the differences observed for genes involved in carbohydrate metabolism could explain the differences in the muscle glycogen content observed from hatching between the two lines [12] or in growth, with pHu- chicks being heavier at this age than pHu + chicks. Recent observations (not yet published) revealed that pHu- chicks are more active during the days after hatching than pHu + chicks, which is consistent with the greater hepatic expression of genes involved in carbohydrate metabolism in this line likely in response to the increased muscle energy need [40].

In addition to being involved in carbohydrate and lipid metabolism, FoxO1 regulates metabolic homeostasis in response to oxidative stress. Alterations in oxidative stress mediated by the accumulation of ROS cause a significant increase in *FOXO1* transcriptional activity with a major impact on the expression of superoxide dismutase (SOD), a key antioxidant enzyme [41]. The antioxidant protection of the chicken embryo during incubation and early postnatal development plays an important role in chick viability [42]. The significantly higher expression of *FOXO1* at E18 and of *SOD2* (mitochondrial form) at E14 and E18 (with a trend at D0) could indicate better removal of ROS and, consequently, better protection of mitochondria against cell death in the liver of pHu- embryos. Uric acid is the main nitrogenous waste product in birds but it is also known to be a potent antioxidant [43]. Glucose transporter 9 (GLUT9) is a key transporter that mediates the uptake of uric acid in hepatocytes [44, 45]. In the liver, expression of the *SLC2A9* gene is positively correlated with the serum uric acid level [46]. Therefore, the overexpression of *SLC2A9* at D8 is consistent with the trend for a higher serum uric acid level (*P* = 0.08) in pHu- chicks.

Hierarchical clustering based on gene expression revealed two subgroups of pHu + chicks: G1 and G2. They mainly differed in the expression pattern of genes involved in energy metabolism, especially in sugar transport (*SLC2A2*, *SLC2A8* and *SLC2A12*), glycogen metabolism (*GSK3* and *PHKA2*), autophagy (*AKT1*, *MTOR*, *RPTOR*, *ULK1*, *ATG4B* and *UVRAG*), mitochondrial activity (*CS* and *COX4I1*) and adenosine monophosphate–activated protein kinase (AMPK)-mediated regulation of energy metabolism (*PRKAA2*, *PRKAB2*, *PRKAG1* and *PRKAG2*) (Additional Fig. 2). G1 was characterised by a higher liver weight and blood triglyceride content and, to a lesser extent, more non-esterified fatty acids and hydroxybutyrate than G2. High triglyceride concentrations support a difference in the availability or use of lipid substrates between G1 and G2, even though they exhibited a similar residual yolk sac weight. As suggested by Ohtsu et al. [47], residual yolk sac lipids are converted to ketone bodies, an essential energy source in newly hatched chicks. Ketone bodies are produced primarily in the liver as a result of incomplete oxidation of long chain fatty acids. They readily serve as fuel for extra-hepatic tissues including the brain, skeletal muscles, intestines and kidneys. At hatching, pHu + chicks had much greater blood creatine kinase levels than pHu- chicks – 4- and almost 8- times higher in G2 and G1, respectively. Creatine kinase is a cytoplasmic or mitochondrial protein abundantly expressed in tissues with a high and fluctuating energy demand such as muscle. It catalyses the conversion of creatine to phosphocreatine, a process that requires the conversion of ATP to ADP [48]. High creatinine kinase activity in hatched G1 chicks could be a sign of a more intense muscle activity, especially of the pipping muscle used to break the eggshell during hatching, or a higher phosphocreatine supply due to low glycogen stores [6, 49]. High creatinine kinase may also reflect intense muscle damage [50], which suggests that the muscle of pHu + chicks would be more solicited and metabolically affected at hatching than that of pHu- chicks, likely because of their low muscle energy reserves [12].

Our study also revealed some differences in gene expression between males and females, despite similar early growth rates. In contrast to the mammalian sex-determining system, in avian species an embryo with two Z chromosomes (ZZ) becomes a male, while an embryo with Z and W chromosomes (ZW) becomes female [51]. As expected, all genes located on the Z chromosome, like *RPS6*, *ACAA2*, *ATG12* and *PRKAA1*, were significantly overexpressed in males but not females at D0 and D8. This finding confirms the results reported by Wang et al. [52] on the catalytic α_1_ subunit of AMPK (*PRKAA1*) in the liver. Other autosome-localised genes were differentially expressed between males and females as soon as they hatched. Interestingly, genes involved in protein synthesis and fatty acid β-oxidation appeared overexpressed in males while genes involved in glycogen metabolism and glucose transport were overexpressed in females. The difference observed in the relative liver weight, in favour of females, could be linked to the different liver chemical composition.

## Conclusions

This study provides a detailed description of the evolution of the different hepatic metabolic pathways during the early development of embryos and post-hatching chicks. Our findings highlight certain metabolic orientations specific to the pHu + and pHu- lines, which are established very early, probably in relation to their different genetic background and nutrient availability in the egg. Indeed, during early *in ovo* development (E12 and E14), pHu + embryos are characterised by greater proteolysis, glycogen degradation, ATP synthesis and autophagy, likely in response to a higher energy requirement than pHu- embryos. The large number of overexpressed genes in pHu- chicks at the end of incubation (E18 to hatching) suggests that they are more prone to mobilise the various metabolic pathways to overcome the huge energy demand for hatching and the slowdown in hepatic metabolism. Moreover, compared with pHu- chicks, pHu + chicks are more heterogeneous, have a lower growth rate during the first week post-hatch and a much higher level of creatinine kinase in the blood at hatching, which could be a sign of greater muscle damage, probably in response to their muscle glycogen deficit.

## Methods

### Animals and sample collection

**Fig. 4:**
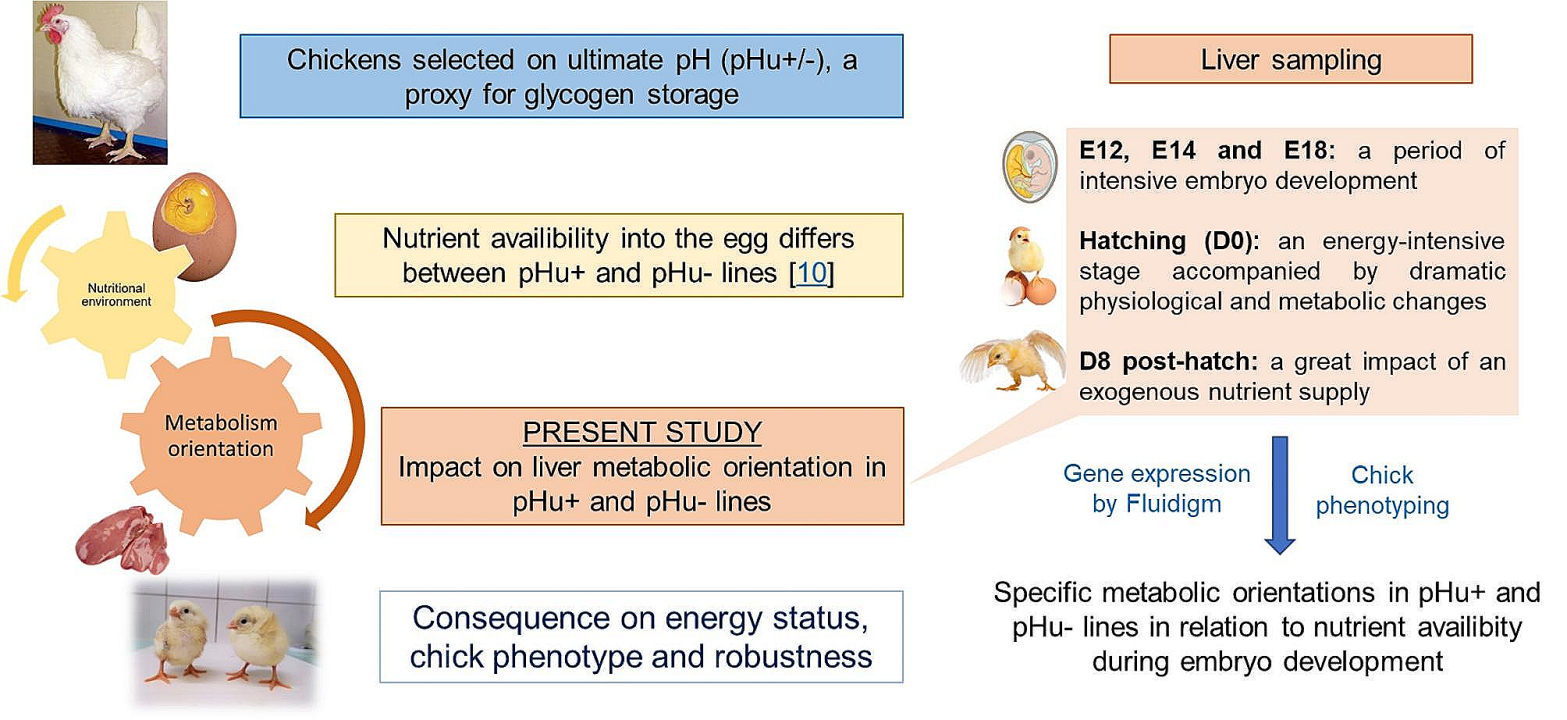
Experimental design of the study. The study was conducted on embryo and chicks from two genetic lines divergently selected for high or low ultimate pH of breast meat (pHu + and pHu-, respectively) to characterize their liver metabolic orientation during embryo development in relation to nutrient availability into the egg

All animal care and experimental procedures for this study were conducted in accordance with current European legislation (EU Directive 2010/63/EU) and were approved by the Ethics Committee for Animal Experimentation of Val de Loire. This ethics committee is registered by the National Committee under the number C2EA 19. Up to D8, chicks were raised under conventional conditions at the PEAT INRAE Poultry Experimental Facility (2018, 10.15454/1.5572326250887292E12). The study was conducted on embryonated eggs from two genetic lines divergently selected for high or low ultimate breast meat pH (pHu + and pHu-, respectively) (Fig. 4). The eggs came from 80 females per line. Fertile eggs were collected for 10 days at the peak of laying (29 weeks of age). Eggs were stored at 16 °C and then incubated under standard conditions at 37.8 °C and 55% relative humidity in the PEAT experimental hatchery. Candling was performed on E7 and E14 to assess early and late embryonic mortality in both lines. The eggs selected for sampling were chosen based on the average weight (± 5%), the day of laying and their family in order to maintain a good representation of each family for each stage. Before and during the incubation period, egg weight was significantly higher in the pHu + line than in the pHu- line (data not shown). Chick liver tissues were collected on E12, E14 and E18. To obtain a sufficient amount of tissue for all the analyses, three animals were pooled for E12 and E14 and two animals were pooled for E18 (*N* = 8 pools per line and per stage). On E18, the remaining eggs were placed in a hatcher (37.7 °C and 65% relative humidity) to take tissue and blood (occipital sinus) samples, at hatching (D0) and at D8. During the first week post-hatch, the reared chicks were fed *ad libitum* with a starter feed. For D0 and D8, tissue samples were collected and analysed individually and considering the sex of the animals (*N* = 20 and *N* = 11 per line respectively at D0 and D8). At D8, chicks were sampled in a fed status. The objective was to consider embryos and chicks with a constant supply of nutrients throughout the analysed period of development (nutrients available *in ovo*, use of the residual yolk sac at hatching and chick feeding). Before freezing in liquid nitrogen and storage at -80 °C, all samples were weighed, except at E12, a stage for which it was difficult to collect the entire liver and *Pectoralis major* muscle.

### RNA extraction

Total RNA was extracted from 30 mg of tissue (liver) using the NucleoSpin® RNA mini-Kit (MACHEREY-NAGEL, Hoerdt Cedex, France) according to the manufacturer’s recommendations. The total RNA concentration was measured with a spectrophotometer (NanoDrop ND 1000) and RNA integrity was confirmed by agarose gel electrophoresis (1%) with the presence of the two major ribosomal RNA bands (28 S and 18 S).

### Quantitative reverse transcription polymerase chain reaction (RT-qPCR) using microfluidic technology (BioMark™ HD)

The mRNA levels of 64 genes involved in protein, carbohydrate, lipid and steroid metabolism; mitochondrial activity; autophagy; thyroid hormone regulation; the antioxidant system; growth; and development were quantified. All primers, listed in Additional Table 1, were tested in the chicken tissue of interest by real-time PCR to determine their efficiency and validate them before further Fluidigm analysis. Some were taken from the literature and others were designed from the chicken (*Gallus gallus*) genome. The choice of primers in the regions of interest was made by using Primer-BLAST and Primer3Plus. Validation of their efficiency ranging from 1.80 to 2.20 was performed using cascade dilution of a pool of complementary DNA (cDNA), and their specificity was confirmed by sequencing the PCR products (GENEWIZ, Part of Azenta Life Sciences, Leipzig, Germany). The quality of total RNA was evaluated by capillary electrophoresis (Bioanalyzer, Agilent Technologies, Les Ulis, France) and conducted on a sampling (total RNA from 12 samples randomly selected by tissue). Total RNA from seven samples were quantified by fluorimetry (Qubit or Varioskan, Thermo Fisher Scientific, Les Ulis, France). A volume of 1 µL of total RNA (100 ng/µL) was reverse transcribed to cDNA with 4 µL of the Fluidigm Reverse Transcription Master Mix kit (Fluidigm P/N 100–6297) (GenomEast platform). The reverse transcription was carried out according to the following programme: 25 °C for 5 min, 42 °C for 30 min and 85 °C for 5 min. Sequencing was performed by the GenomEast platform, a member of the ‘France Génomique’ consortium (ANR-10-INBS-0009). PCR was conducted in integrated fluidic circuits (IFCs) of the Dynamic™ Arrays (96.96 IFC). Due to the low final PCR volume (6–9 nL), target genes were pre-amplified (PreAmp Master Mix kit, STA mix, Fluidigm P/N 100–5744). This pre-amplification consists of multiplex PCR with a reduced number of cycles (10–20 cycles) using a pool of all primer or probe pairs that will be used later in the BioMark™ HD. The pre-amplification was carried out according to the following programme: 95 °C for 2 min, followed by 14 cycles at 95 °C for 15 s and 60 °C for 4 min. Unused primer residues were digested with exonuclease I (20,000 U/mL, M0293L, New England BioLabs) at 37 °C for 30 min and then at 80 °C for 15 min. Each final product was diluted 1:5 by adding reduced TE buffer (10 mM Tris, pH 8.0 + 0.1 mM EDTA, P/N 12090-015, Invitrogen). To prepare the primer plate, a mix containing 371 µL of 2X Assay Loading Reagent (P/N 85,000,736, Fluidigm) and 296.8 µL of reduced TE buffer was prepared. Each well of the assay plate received 6.3 µL of this mix along with 0.7 µL of primers (forward and reverse, 50 µM). Negative controls, without primers, received 0.7 µL of reduced TE buffer and 6.3 µL of Dilution Mix. For each sample plate, a mix containing 371 µL of 2X SsoFast Evagreen Supermix with Low ROX (172–5211, Bio-Rad Laboratories, Marnes la Coquette, France) and 37.1 µL of 20X DNA Binding Dye sample reagent (P/N 100–3738, Fluidigm) was prepared. Each well of the assay plate received 3.85 µL of this mix and 3.15 µL of pre-amplified cDNA. Negative controls, without cDNA, received 3.85 µL of mix and 3.15 µL of reduced TE buffer. After vortexing and centrifugation, the Dynamic™ array was primed and loaded with pre-amplified amplicons and primer pairs (5 µL per well) by using an appropriate IFC controller (IFC Controller HX, 96.96 Dynamic Array IFCs). For detailed instructions for injecting the control line fluid, see the Control Line Fluid Loading Procedure Quick Reference (68,000,132). PCR was performed using the GE Fast 96 × 96 PCR + Melt v2 thermal cycler protocol (thermal mix: 70 °C for 2400 s, 60 °C for 30 s; hot start at 95 °C for 60 s; and 30 cycles of 96 °C for 5 s, 60 °C for 20 s and 60 °C for 3 s). Real-Time PCR Analysis software v.3.0.2 or later and BioMark HD Data Collection software v.3.1.2 or later are required for this protocol.

### Data preprocessing and gene expression analysis

The first part of the analysis was to visualise the cycle threshold (Ct) values as well as the melting curves with the Fluidigm real-time PCR analysis software. Genes with very high (Ct < 2.5) or very low (Ct > 24) expression were removed from the analyses, along with genes for which an aberrant melting temperature (Tm) or multiple amplicons (dimers of primers or non-specific products) were observed in the melting curves. First, the expression data were normalised using interplate calibrators. The delta Ct (ΔCt) was then calculated according to the following formula:

$$\Delta C{t_{target}} = {\text{ }}C{t_{control}}-{\text{ }}C{t_{sample}}$$

Relative gene expression = 2^ΔCt target^ / normalisation factor.

Housekeeping genes were selected and the normalisation factor calculated using the geNorm software (3.5). The housekeeping genes used to normalise the expression of the target genes (line and sex effects within developmental stage) were *DIO1* and *HADH* at E12; *GAPDH* and *SLC2A2* at E14; *PRKAG1* and *YWHAZ* at E18; *RPTOR* and *ATG4B* at D0; and *ANT3* and *COX4I1* at D8. As embryogenesis and early life are dynamic periods of development, housekeeping gene expression fluctuated between the different stages, which explains why the expression data were not normalised. However, interplate normalisation using calibrators provided a relevant argument to free ourselves from target gene normalisation.

### Biochemical assays

The serum glucose (Glucose GOD-POD, ref 981,780, Thermo Fisher Scientific, Dardilly, France), uric acid (Uric Acid AOX, ref 981,788, Thermo Fisher Scientific, Dardilly, France), triglyceride (ref 981,786, Thermo Fisher Scientific, Dardilly, France), non-esterified fatty acid (NEFA-HR(2), FUJIFILM, Sobioda, Montbonnot Saint Martin) and hydroxybutyrate (RANBUT, RB 1007/MD, RANDOX, Roissy) concentrations; the serum total antioxidant status (TAS, NX2332, RANDOX, Roissy); the creatine kinase activity (CK-NAC, ref 92,307, BIOLABO, Abliance, Compiègne, France); and the liver glycolytic potential (Glucose HK, ref 981,779 and L-lactic acid, ref 984,308, Thermo Fisher Scientific, Dardilly, France) were measured spectrophotometrically using commercial kits.

### Statistical analysis

For each pHu line, heatmaps were produced in two steps. The first step was to perform hierarchical clustering of all genes and samples to check the classification of stages (E12, E14, E18, D0 and D8) and to identify any outliers. This approach also revealed two groups (G1 and G2) at D0 in the pHu + line. In the second step, developmental stages were ordered and hierarchical clustering was performed on genes only. At D0 and D8, samples were ordered according to sex. G1 and G2 obtained at D0 in the pHu + line were kept as obtained in the first step. In each group, males and females were pooled together. For graphical representations, the variables were log2 transformed, centred and reduced. Heatmaps were obtained with the R software (4.2.2) using correlation as a distance measure (Pearson) and the ward.D2 method as the aggregation criterion for clusters. The pheatmap (1.0.12), grDevices (4.1.2) and Stats (4.1.2, hclust function) packages were also used. Average expression profiles were generated by using the cutree function.

For relative expression data, serum assays and weight parameters, main effects and interactions were analysed by one- or two-way analysis of variance (ANOVA) (*P* ≤ 0.05). Depending on the number of samples, means were compared by Student’s t-test or Tukey’s test (XLSTAT 2020.5.1 and StatView 5).

### Electronic supplementary material

Below is the link to the electronic supplementary material.

Supplementary Material 1

Supplementary Material 2

Supplementary Material 3

Supplementary Material 4

## Data Availability

All analyzed data from the fluidigm study have been deposited on https://zenodo.org/records/10149721 (doi 10.5281/zenodo.10149721).

## Abbreviations

G1: Group 1 (pHu+)
G2: Group 2 (pHu+)
CK: Creatine kinase
T3: Triiodothyronine
T4: Thyroxine
T2: Diiodothyronine
IGFs: Insulin-like growth factors
ADP: Adenosine diphosphate
ATP: Adenosine triphosphate
AMP: Adenosine monophosphate
AMPK: AMP-activated protein kinase
ROS: Reactive oxygen species
GLUT9: Glucose Transporter 9
mRNA: Messenger ribonucleic acids
cDNA: Complementary deoxyribonucleic acid
PCR: Polymerase Chain Reaction
IFCs: Integrated fluidic circuits
Ct: Cycle threshold
Tm: Melting temperature
