# Supplementary material for: Ontogeny of hepatic metabolism in two broiler lines divergently selected for the ultimate pH of the Pectoralis major muscle
Source: BMC Genomics. 2024 May 2;25:438. doi: 10.1186/s12864-024-10323-0 (PMC11067279; doi:10.1186/s12864-024-10323-0)
Supplement: Supplementary file 1 — Supplementary Material 1 [file 12864_2024_10323_MOESM1_ESM.docx]

**Additional Fig. 1** Network representing protein–protein associations of interest at hatching.

The proteins included in the network correspond to genes for which there was a peak in expression or a transient increase at hatching in both lines. The thickness of the lines indicates the confidence of the associations. Fine line = between 0.2 and 0.4; medium line = between 0.4 and 0.7; thick line = between 0.7 and 0.9; extra-thick line = more than 0.9. When the three-dimensional structure of the protein is known or has been predicted, it appears in the node that is specific to it. Red nodes = glycogen metabolism, yellow nodes = macroautophagy, green nodes = glycogen recognition site of AMP-activated protein kinase, dark blue nodes = cellular response to nutrient levels and light blue nodes = reactive oxygen species metabolic process. SLC25A6 = ANT3 and RPS6KB1 = P70S6K.
