# Supplementary material for: Ontogeny of hepatic metabolism in two broiler lines divergently selected for the ultimate pH of the Pectoralis major muscle
Source: BMC Genomics. 2024 May 2;25:438. doi: 10.1186/s12864-024-10323-0 (PMC11067279; doi:10.1186/s12864-024-10323-0)
Supplement: Supplementary file 2 — Supplementary Material 2 [file 12864_2024_10323_MOESM2_ESM.docx]

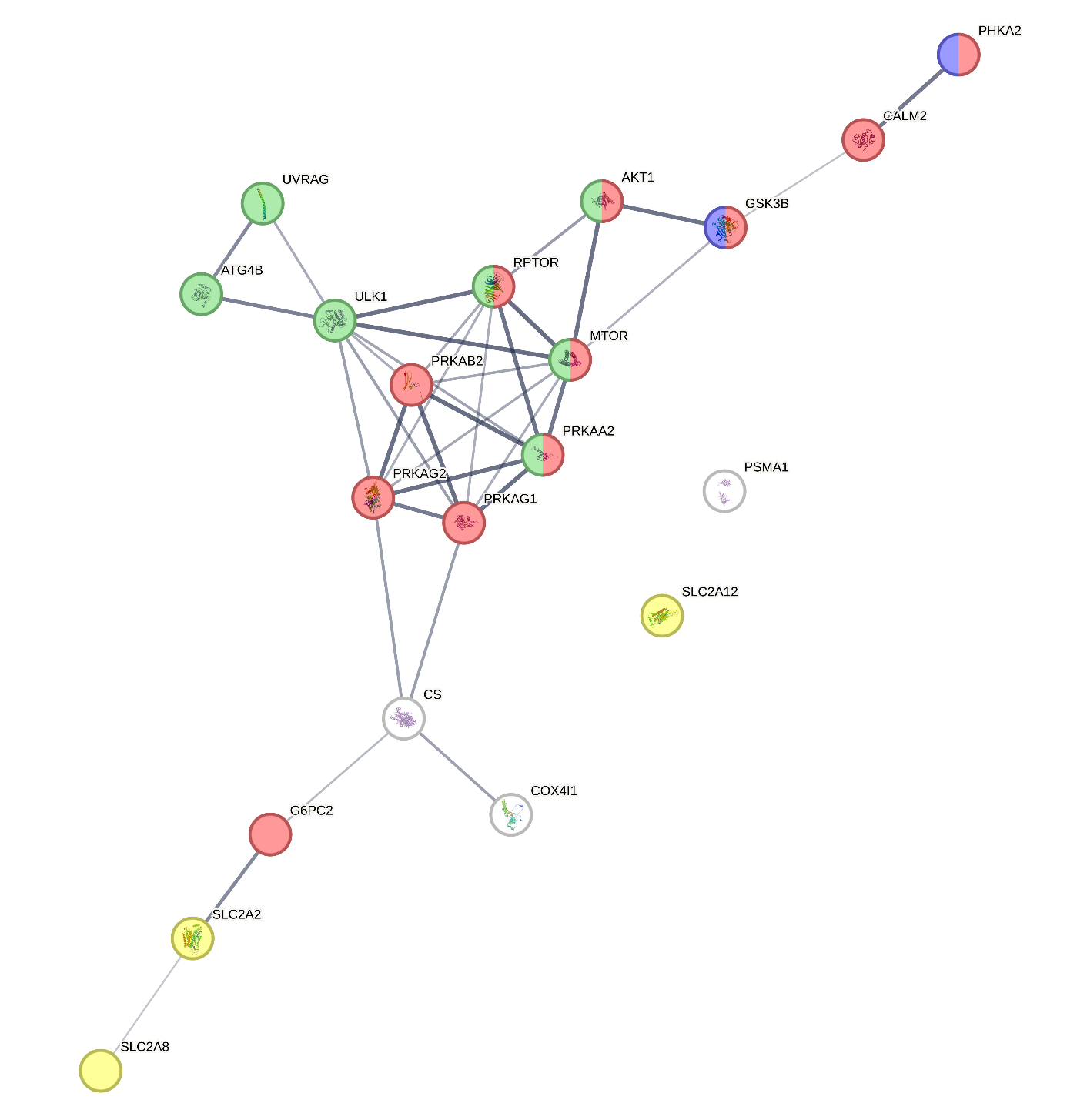


**Additional Fig. 2** Network representing protein–protein associations of interest between groups 1 and 2 (the pHu+ line).

The proteins included in the network correspond to genes for which there were visual differences in the pHu+ line between group 1 (G1) and group 2 (G2) at hatching. The thickness of the lines indicates the confidence of the associations. Fine line = between 0.2 and 0.4; medium line = between 0.4 and 0.7; thick line = between 0.7 and 0.9; extra-thick line = more than 0.9. When the three-dimensional structure of the protein is known or has been predicted, it appears in the node that is specific to it. Red nodes = insulin pathway, yellow nodes = sugar transporters, green nodes = autophagy and blue nodes = glycogen metabolism. CALM2 = PHKD.
